# The Best Means of Preserving the Eye-Sight of Dental Practitioners

**Published:** 1873-01

**Authors:** I. Williams


### The Best Means of Preserving the Eye-Sight of Dental Practitioners.

BY I. WILLIAMS.

*Read Before the Ohio State Dental Society, at the Seventh Annual Meeting, December*, 4, 1872.

No subject can be of more practical importance to the dental practioners than the preservation of distinct vision for the longest possible period of time.

The general medical practioner may pursue his vocation with a fair degree of success, and satisfaction to himself and his patients, even after the sight has become very greatly impaired by age or otherwise.

The minister may pursue his calling with brilliant success though total darkness enshroud him. Who has not read the story of "the blind preacher," and the magical power of his eloquence?

With the dentist the case is entirely different. To him a good clear eye is of primary importance, and without it every accomplishment in knowledge and skill will be useless. How we may preserve the functions of this most important and wonderful organ should engage our most careful attention.

Any theory of preservation that ignores or in any way overlooks the general health, and vital and nervous force, is, sadly defective if not entirely erroneous. It is true that the eye may be injured by an imprudent act or course of conduct while the general health may be good; but it is not true that a course of living which tends to break down the general health, and which cripples the vital functions, can be pursued without serious injury to the eye as well as to every other organ of the body. Chronic derangement of the stomach produces that very troublesome disease called *Muscœ volitantes* in which the patient sees flies, motes or little clouds that seem to be moving before his eyes. Correct the derangement of the stomach by right living, and the flies and motes will disappear.

Dentists have had frequent opportunities to demonstrate that a diseased condition of the mouth and teeth may produce various forms of disease of the eye. As well might we expect to find the branches and leaves of a tree in a vigorous and flourishing condition while the trunk and roots are partially dead and dying.

Whatever then will tend to build up a vigorous and healthy constitution, tends directly to preserve and prolong the sight.

The habit of early rising is said to be of great advantage in preserving and prolonging distinct vision. It is a well established truth that sudden changes from darkness to light or from light to darkness are injurious. The individual who rises late and suddenly emerges into the clear strong light of day, certainly inflicts a serious injury upon the delicate structures of the visual organs. Were the sun to burst suddenly upon us in his meridian splendor, instead of making his appearance as he does, universal blunders would soon ensue. The design of nature may generally be inferred by the way in which she manifests herself. What can more clearly indicate design than the very gradually increasing light of day?

An octogenarian eminent for his good common sense, and who never had occasion to wear glasses, attributes the remarkable preservation of his sight to his constant habit of early rising.

The dental practitioner should be careful to have his operating window facing the north. He will then not be annoyed by the sun shining in his face, or by its rays being reflected from his instruments into his eyes.

Perhaps no one thing more generally and seriously injures the eye of the dentist than straining to secure clear and exact vision. This practice persisted in will sooner or later ruin the best eye.

Let it be settled that by position of patient or such other means as may be necessary, you will secure a clear and distinct view of the point to be operated upon. An intelligent patient of mine once observed, after I had placed him in a position not the most agreeable, "I can stand it; it is only an occasional thing with me, with you it is constant." That remark has done me much good.

Another difficulty peculiar to dentists is the necessity of overtaxing the powers of the eye and nervous system in the performance of the large and complicated operations they are now called upon to make. Holding the eye upon a single point till there is a sensation of exhaustion and indistinctness of vision, is exceedingly injurious. Since the introduction of the rubber dam this inconvenience may be very greatly overcome. By stopping in the midst of these large operations, and changing the position and letting the eye rest on other objects, the difficulty may be relieved if not entirely overcome. An operation should not be commenced unless the dentist feels certain of its completion before the light grows dim in the evening.

Some dentists, by a foolish and very injudicious habit of bringing the eye unnecessarily near their work, have become permanently near-sighted. This point should be carefully guarded. Indeed every man whose occupation requires very fine sight is liable to the same difficulty, and care should be taken to correct the eye by viewing distinct objects as much as possible.

The habit of looking through eye-glasses having short focal distances with one eye tends to shorten the focal distance of that eye. It is often necessary for the dentist to use a glass that magnifies, but would it not be better to use spectacles that magnify, then both eyes will be brought under the same optical influence. A jeweler of my acquaintance told me that he had nearly ruined his eyes by the constant use of a single glass.

The eye is very greatly effected by mental inflence. Joy and grief plays no unimportant part in preserving the natural form and function of the eye, or of flattening its globe and impairing vision. The former aids wonderfully in perpetuating distinctness of vision to a green old age, while the later exerts a potent influence in causing "those that look out of the windows to be darkened." The dentist who by diligence has made himself so proficient in his specialty as to give good and permanent satisfaction to his patients, and who by his honorable and gentlemanly deportment has secured the good opinion of his patrons and of the community; who by his industry and frugality is able to gain a competence for the comfortable support of himself and family, and who by his kindness and goodness of heart has made a little paradise out of his home, to which he may retire for enjoyment and recuperation—has done much toward putting off a little longer the evil day of spectacles.

The eye should be kept clean by frequent bathing. Immersing the face in clear water not too cold, and rinsing in the morning, then opening and shutting the eyes a few times, is a good practice. The eye is thus cleaned, and if there be acrid humors they are neutralized.

The eye needs and should have an abundant supply of pure air. No pestiferous odors should be allowed to remain in the dental office. Those who live in dark, damp apartments, where the air is loaded with smoke and other impurities, are usually troubled with sore eyes and indistinctness of vision.

Whether these effects are produced by first undermining the general health I cannot say.

All good medical writers regard tobacco smoke as very injurious, especially where there is a tendency to inflamation. Many of the non-smoking members of this Association have realized the terrible effects of tobacco smoke, not only upon the delicate structures of the eye but upon the whole man, soul and body, during the meeting of our Association.

Some diversity of opinion exists as to the propriety of commencing the use of glasses, when the power of distinct vision at the natural or usual focal distance ceases. Some contend that if at this period, which usually occurs at from forty to forty-five, the eye is compelled to do duty without the aid of artificial helps, nature under the pressure will soon restore the form of the optical organs, so that sight at the ordinary distance will be as good as ever. Nature, it is contended, is equal to any emergency.

But however beautiful the theory, facts do not seem to corroborate its truthfulness. As well might we assume that continued persistence in masticating food on the gums after the second set of teeth have been removed would most assuredly devolve a third set. The course proper to be pursued seems simply to be this: When at the period in life above referred to, difficulty is experienced in the perception of small objects, when in the careful scrutiny of minute details the light seems insufficient, and the object blurred and indistinct, and when continued effort increases the difficulty, and causes pain in and above the eye, we should be admonished that nature, much abused, is beginning to succumb, and however much we may apply the lash and spurs, she will not respond as aforetime. The demands of nature are stern and relentless, and penalties must be paid.

Recourse to glasses is the only remedy. There may be however, cases where rest and diversion from business is all that is needed.

The rules for selecting glasses, as well as the treatment of the diseases to which over-worked eyes are liable, we leave for the Society to discuss.

